# Sugar responsiveness could determine foraging patterns in yellowjackets

**DOI:** 10.1038/s41598-023-47819-w

**Published:** 2023-11-22

**Authors:** Maité Masciocchi, Analía Mattiacci, José M. Villacide, Micaela Buteler, Agustina P. Porrino, Andrés S. Martínez

**Affiliations:** 1 Grupo de Ecología de Poblaciones de Insectos, IFAB - Instituto de Investigaciones Forestales y Agropecuarias Bariloche (INTA - CONICET), Bariloche, Argentina; 2 INIBIOMA - Instituto de Investigaciones en Biodiversidad y Medioambiente (CONICET - UN Comahue), Bariloche, Argentina

**Keywords:** Ecology, Behavioural ecology, Invasive species

## Abstract

Sympatric-related species often exhibit resource partitioning. This can occur through different mechanisms, such as behavioral, morphological, and sensory variations, leading to qualitative, temporal, or spatial differences in resource exploitation, such as consuming different types of food. Sensory-based niche partitioning could be the underlying mechanism through which closely related species effectively reduce niche overlap. Here we ask whether variations in sensory responses to carbohydrates could reflect differences in the foraging patterns of two *Vespula* species present in Patagonia. For this, we established (i) the response thresholds toward carbohydrate solutions of foraging *V. germanica* and *V. vulgaris* in the laboratory, (ii) the sugar concentration of foraged carbohydrates in the field, and (iii) possible effects of incoming sugar concentration and performance at individual and colony levels. Results indicate a higher sucrose response threshold in *V. germanica* than *V. vulgaris*. Field results indicate that higher carbohydrate concentrations foraged by *V. germanica*, with 57% of *V. germanica* foragers returning with concentrations above 50% w/w, while only 23% of *V. vulgaris* foragers did so. These differences in sucrose sensitivity and foraging patterns positively correlate with colony size, irrespective of the species. Our results suggest that competition could be reduced in these closely related invasive social wasp species through sensory differences in their sugar perception levels, which would lead to them foraging different carbohydrate sources. This study suggests that sensory niche partitioning could promote species coexistence in these social wasps.

## Introduction

According to the competition-relatedness hypothesis, when two closely related organisms coexist, they are more likely to compete and exclude one another because they occupy similar ecological niches and have similar life-history traits. Nevertheless, it is not unusual to find ecologically similar species inhabiting the same community, indicating the presence of strategies that allow coexistence^1^. If behavior and resources are limited, and similar feeding patterns overlap, competition may become the dominant strategy, leading to the displacement of one of the species^2,3^. Sympatric-related species often exhibit resource partitioning, which occurs when they display differences in resource exploitation. This can occur through distinct mechanisms, such as behavioral, morphological, and sensory divergence, leading to qualitative, temporal, or spatial heterogeneity in resource exploitation, such as consuming different types and qualities of food or utilizing different areas/sources of the environment. Darwin's finches are the classical textbook example of how resource partitioning through morphological distinctness can modulate habitat use and hence competition. The contrasting beak sizes and shapes of 15 finch species, inhabiting the Galapagos archipelago result in contrasting diet differences^4,5^. Other morphological differentiation consists of the proboscis shape of bumblebees that determines the flowers from which they extract nectar^6,7^.

Sensory-based niche partitioning has not been studied as frequently as other mechanisms such as variation in morphology of temporality. Studies in bats suggest an important role of sensory ecology in the structuring of these vertebrate communities, where the auditory responses of different bat species, result in dissimilar prey detection that minimize diet overlap^8–11^. Such differences have also been found to reduce competition in various insect groups. For instance, bumblebee communities have been shown to segregate along a gradient of light intensity due to differences in visual traits: species displaying a higher investment in light sensitivity were observed to forage under dimmer light conditions when compared to species with a lower eye parameter, identifying a linear correlation between eye parameter of the species and their realized niche optimum^12^. Sensory niche partitioning seems to take place in Drosophilid flies, where visual and olfactory differences in *D. subobscura* and *D. pseudoobscura* result in niche partitioning and relaxed competition helping them coexist ^13^. Additionally, in honeybees and bumblebees, where both forage for pollen, it has been observed that bumblebees tend to collect pollen with higher protein content and more essential amino acids than the one collected by honeybees, which could be guided by differences in gustatory thresholds ^14^.

Yellowjacket wasps (*Vespula* spp.) are recognized for their invasive success^15,16^. These eusocial wasps have rapidly spread to several regions of the world over the past century^17,18^. In Argentina, two species have been established successfully: *Vespula germanica* was first reported in the 1980s, while *Vespula vulgaris* was detected in 2010^19,20^. Both species pose significant problems for human and economic activities due to their dangerous stings, rapid population growth, adaptable behaviors, generalist diets, and detrimental effects on ecological and productive systems^21,22^.

*Vespula germanica* and *V. vulgaris*, both sympatric and native to Eurasia, coexist in invaded regions such as Argentina, Chile, Australia, and New Zealand^18^. Despite their similar biology, these generalist and opportunistic wasp species may have developed strategies to minimize competition in their native range, affecting their shared success in invading countries. Examples from New Zealand and Argentina (Patagonia) indicate that these wasps spatially partition resources. *Vespula vulgaris* tends to forage in shrubland at mid canopy-height levels, while *V. germanica* is more commonly found at ground level^23,24^. Moreover, on the northeast coast of New Zealand, differences in the diet of *V. vulgaris* and *V. germanica* have been observed across different seasons^25^. Similarly, studies conducted in Argentina have demonstrated that *V. germanica* actively avoids visual and odor cues from *V. vulgaris*, as observed in bioassays with free-flying wasps approaching protein baits^26^. The coexistence of these generalist invaders in certain locations may be facilitated by partitioning resources through differences in foraging behavior.

Little emphasis has been placed on investigating interspecific variations in the sensory mechanisms employed by wasps for food detection, or their significance in facilitating resource partitioning and, consequently, promoting coexistence among species. *Vespula spp.* wasps collect carbohydrates from diverse sources such as insect honeydew, honey produced by *Apis* bees, nectar, and human food. These carbohydrates are primarily used as a source of energy and for thermoregulation in wasp^27,28^. Variation in carbohydrate concentration among different sources is common, and regional studies in Patagonia have determined that flower nectar varies considerably, ranging from 12 to 50% w/w^29^. Therefore, this type of resource could potentially be exploited differently by related co-occurring species. Variations in sensory responses to different resources during food foraging can influence the likelihood of exploiting a certain resource. In this context, behavioral bioassays have been developed to assess the sensitivity to sucrose solutions by measuring the proboscis extension response (PER) in bees since this is a response triggered when the antennae encounter the sucrose solution^30^.

Here we ask whether variations in carbohydrate sensory responses could reflect differences in the foraging patterns of two *Vespula* species present in Patagonia. Our objective was to describe carbohydrate responsiveness and foraging patterns of *V. vulgaris* and *V. germanica* under the hypothesis that sensory differences could modulate niche partitioning. Specifically, we established (i) in the laboratory, the response thresholds toward carbohydrate solutions of foraging worker wasps of *V. germanica* and *V. vulgaris* and (ii) in the field, the quality (i.e., sugar concentration) of foraged carbohydrates and (iii) its correlation with individual and colony performance. Our working hypothesis was that different carbohydrate foraging habits, as determined by physiological response thresholds to carbohydrates, may be one of the mechanisms that enables their coexistence, without affecting performance.

## Methods

The study was carried out by contemplating two distinct scales. We first measured individual sensory responses of *V. germanica* and *V. vulgaris* foragers toward sucrose solutions in the laboratory via the Maxillae-Labium Extension Response (MaLER) technique^31,32^. Second, we measured the sugar concentration of carbohydrates carried by foragers of both species under natural field conditions and evaluated whether there was a correlation between incoming carbohydrate concentration and nest and individual performance.

### Carbohydrate response thresholds

#### Individuals

Wasps used in this study were obtained by excavating active nests (*V. germanica: n* = 6 and *V. vulgaris: n* = 4) in the region of San Carlos de Bariloche, Argentina (41°08' S and 71°18' W) during February and March 2023. Subterranean nests were anesthetized with ethyl ether (98% purity; Sigma Aldrich, St. Louis, MO, USA) and excavated. Immediately after removing the nests, they were placed individually in experimental boxes and transported to the field station at IFAB and kept under natural feeding regime and ambient conditions, until the end of the experiments. Each experimental nesting box consisted of a square aluminium box (30 cm per side) with a transparent plastic tube (25 cm in length × 2.5 cm diameter) to allow free movement of wasps. The top side of the square box was covered with a removable lid, under which a glass top was placed to observe colony development inside the container. The remaining walls were isolated with high-density Styrofoam (2.5 cm thick) for thermal insulation. Preliminary studies showed that wasps continue their normal activities under controlled conditions (Martínez et al. 2021).

Individuals used in bioassays were captured upon their return to experimental nests from foraging trips between 15 and 30 March 2023 on sunny days from 09:00 am to 11:00 am. It’s important to note that individuals of both species were captured and tested simultaneously each day. A cotton wool plug was placed at the nest entrance and for two minutes all individuals entering the tube (i.e., workers returning from a foraging trip) were captured and transferred to a plastic container (50 ml) with a lid. Once captured, wasps were taken to the laboratory, anesthetized with a short burst (5 s) of CO_2_ and individually, light pressure was applied to the abdomen using a flat rod, to extract any liquid food from the digestive system. Even though immobilization of insects with CO_2_ is a common practice^33^, previous studies indicate that exposure to this gas can alter the behavior and physiology of other insect species^34^, but the specific effects on *Vespula* spp. are mostly unknown. In this context, it’s important to mention that CO_2_ was administered in the same manner to all individuals in the experiment, therefore any adverse effects were equal to both species. The concentration of the regurgitated liquid droplet was subsequently measured using a handheld refractometer (0–80 ^o^Brix, Alla France). It’s important to note that the ^o^Brix scale is equivalent to % w/w solids in liquid. This implied the assumption that the contents obtained from the expelled crops were considered to be carbohydrates diluted in water^35^. We used % w/w as a proxy for carbohydrate quality since it is the most frequently reported quantitative measurement of nectar quality in the literature^36^. Wasps that did not regurgitate or those that regurgitated a droplet with a concentration of < 1% w/w were excluded from the experimental procedure due to containing only sugar traces, thus considered to be water carriers, and not involved in carbohydrate foraging. Subsequently, each anesthetized wasp was carefully immobilized within a modified Eppendorf tube (2 ml), restricting movement to all body parts except the antennae and mouthparts. After a period of acclimatization in a dark room at 25 ± 1 °C for 75 ± 15 min, wasps were given ad libitum water in order to discard confounding effects due to thirst later in bioassays.

#### MaLER experiments

To determine the sucrose response threshold of wasps, the MaLER technique was used. This experimental approach relies on stimulating sequentially the wasps' antennae with seven carbohydrate solutions of increasing concentration (i.e., trial: 0.01, 0.1, 1, 3, 5, 10, 50% w/w) while evaluating the extension of the maxillae-labium apparatus (response). Antennal stimulation was done with wooden toothpicks soaked in the respective sucrose solutions. Each stimulation lasted 3 s, and prior to each, the wasps were provided with water (via a toothpick) to discard sensitization or habituation effects. A single wasp was tested at a time, with a 4-min inter-stimulus interval. The MaLER response was assessed and scored as either “1” if the response was observed, or “0” if no movement was detected. Wasps that failed to exhibit a complete response to any stimulus were excluded from the analysis. It is important to note that none of the tested wasps responded to all the stimuli, suggesting a state of overexcitation. Consequently, the criterion for complete non-responsiveness was not applied in this study. Furthermore, wasps that did not respond to the gustatory stimulus despite showing a response to water were also excluded, assuming a low level of feeding motivation. Assays were carried out from 11 am to 5 pm.

#### Carbohydrate foraging

The field study was carried out under natural conditions in the region of San Carlos de Bariloche, Rio Negro, Argentina, where *V. germanica* and *V. vulgaris* wasps coexist and are abundant. The study area encompassed ca. 150 ha. consisting of mixed shrubland and scattered trees (41° 8´ 22´´ S and 71° 2´ 7´´ W). The mean temperature in the region is 8.5 °C and the mean annual rainfall is 1100 mm concentrated during winter. The field studies were carried out from March 8th to April 13th of 2022 on sunny and windless days.

Wasp nests were located, georeferenced and the species were confirmed by observing the characteristic marks on the faces of 3 individuals captured at nest entrances. At each sampling date individuals of both species were measured. First the traffic rate of nests was determined by counting the number of individuals entering or leaving the nest in one minute (Malham et al. 1991). Second, to measure the quality of foraged carbohydrates, nest entrances were blocked with a small cotton wool ball to capture only incoming wasps (and not those outgoing) via a hand-held battery-operated aspirator (Einhell TE-VC 18/10 Li-Sol) for 3 min. After this, individuals were anesthetized with CO_2,_ and their abdomens were gently pressed with a flat rod to promote the regurgitation of liquids. Wasps were given a 5-min recovery period before the abdomen was pressed. This liquid was collected in a micro-capillary tube (5 µl) and the drop was transferred to the handheld optical refractometer (0–80 ^o^Brix, Alla France), to determine the sugar concentration of the liquid. The number of individuals captured without liquid in their crops was also recorded.

To evaluate the performance at individual and colony levels, we used nest size (number of queen and worker cells per nest) and the size/weight ratio as an indication of nutritional levels. To measure this, all nests were carefully excavated (as described above) at the end of the season in mid-April and immediately after stored in a freezer (− 8 °C) for at least 24 h. Queen cells are easily recognized because of their larger size vs. worker cells. Before freezing the nest, 20 individuals per caste/nest were collected, and individual measurements of thorax width and weight were taken. Thorax widths were measured with a digital calliper (VWR, 0.01 mm), and weight with a digital scale (Ohaus Pioneer™, 0.0001 g.). Based on these indicators of size and weight, an index of nutritional status was estimated for gynes and workers (nutritional index: weight/thorax width).

### Data analysis

#### Carbohydrate response thresholds

A generalized linear mixed model (GLMM) with binomial distribution and MaLER as the response variable, was fitted to evaluate the response thresholds towards carbohydrate solutions in foraging wasps. Fixed explanatory variables included "species” and "trial” while random effects were accounted for by "individual" and "nest". Post hoc pairwise comparisons between species were conducted using paired comparisons. The GLMM was fitted using the ‘glmer’ function from the ‘lme4’ package (Bates et al. 2014), while the ‘emmeans’ function from the package of the same name was applied for conducting the contrasts (Lenth 2022). All analyses were performed using R software v.4.3.1^37^.

#### Carbohydrate foraging

To assess differences in crop content concentration between *V. germanica* and *V. vulgaris*, a GLMM with normal distribution was carried out with “crop carbohydrate concentration” as the response variable, “species” as the main fixed effect and “nest”, “date” and “hour” as random variables. Differences between species in the proportions of foragers carrying “water” (% w/w < 1), “sugary liquid” (% w/w ≥ 1) and “empty” wasps were evaluated using the proportions of each capture date. The effect of factors was assessed using mixed-effects multinomial logistic regression with a multinomial error structure, where the saturated model included “species” as a fixed factor, and “nest” and “day” as random factors. Post-hoc pairwise comparisons between species for each crop content were conducted using paired comparisons. The models were fitted using the 'mblogit' function from the ‘mclogit’ package (Elff 2022), while the ‘emmeans' function from the package of the same name was used for conducting the contrasts (Length 2022). An analysis of variance (ANOVA) was used to compare the saturated model with the null model. We chose the model selection procedure to assess the significance of the 'species' factor and packages in the R environment for modelling multinomial distributions and directly testing the effects of factors.

In order to examine the effect of incoming sugar concentration on nest and wasp parameters, a Standard Least Squares analysis was performed. The model was constructed with “average concentration of incoming carbohydrates/nest” and “species” as fixed variables, contemplating their interaction. We used, “number of worker cells'', “number of gyne cells” and “number of total cells in nest (i.e., worker cells + gyne cells)” and “worker nutritional index” and “gyne nutritional index” as responses.

To account for differences in crop content concentration, only those individuals with crop contents with ≥ 1% w/w were considered in the analysis. For each nest, sugar contents were measured at two dates through the season between 8 March and 13 April and averaged to obtain one indicator of sugar concentration/nest corresponding. All analyses were performed using R software v.4.3.1^37^.

### Significance statement

According to the competition-relatedness hypothesis, when two closely related organisms coexist, they are likely to compete because they occupy similar ecological niches. Nevertheless, it is common to find similar species inhabiting the same community, indicating strategies that allow coexistence. Carbohydrates are crucial in contributing to the ecological success insects. Here, we ask whether variations in carbohydrate sensorial thresholds could modulate foraging of two wasps present in Patagonia. We evaluated carbohydrate responsiveness and foraging patterns of *Vespula vulgaris* and *V. germanica* under the hypothesis that sensorial differences modulate niche partitioning. Our results suggest that competition could be reduced in these social wasps through sensorial differences in sugar perception. This study suggests that sensorial niche partitioning could modulate species coexistence in social wasps.

## Results

### Carbohydrate response thresholds

**Figure 1:**
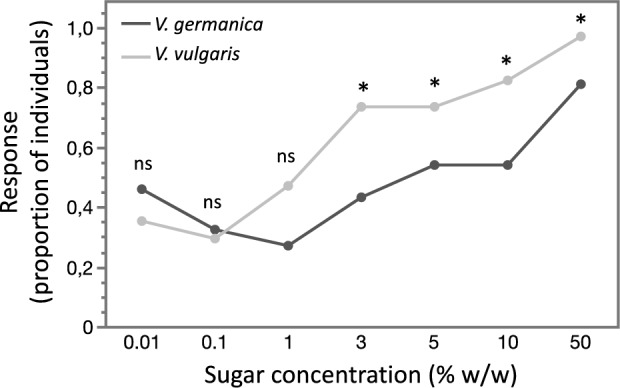
Response levels of *Vespula germanica* and *Vespula vulgaris* workers towards sucrose solutions of increasing concentrations. Responses measurements were done on workers captured at nest entrances with liquid crop loads (≥ 1% w/w) and subjected to the Maxillae-Labium Extension Response technique. Statistical differences were observed as of concentrations ≥ 3% w/w, with a lower proportion of *V. germanica* individuals responding when stimulated (ns = p > 0.05, * = p ≤ 0.05).

A total of 89 wasps were captured (*V. germanica* = 45 and *V. vulgaris* = 44). Of these, 17 individuals did not carry any liquid load (*V. germanica* = 8; *V. vulgaris* = 9), only 5 V*. vulgaris* wasps carried water (% w/w < 1), while all other individuals carried sugary liquid (% w/w ≥ 1) in the crop (*V. germanica* = 37 and *V. vulgaris* = 34) and were tested for MaLER. Significant differences in the response levels of carbohydrates foragers toward sugar solutions of increasing concentration were detected between *V. germanica* and *V. vulgaris* (species:trial = 0.004, Table S1). As sugar concentration increased across trials, response levels of *V. germanica* remained consistently equal to or lower than those of *V. vulgaris*. In *V. vulgaris*, responses started to increase at lower concentrations, with a statistically significant rise in the proportion of individuals responding to the solutions as of 3% w/w vs. *V. germanica* (p 0.01% w/w: 0.41, p 0.1% w/w: 0.63, p 1% w/w: 0.26, p 3% w/w: 0.01, p 5% w/w: 0.01, p 10% w/w: 0.002, p 50% w/w: 0.027; Fig. 1; Table S2).

### Carbohydrate foraging (field study)

A total of 1059 (363 *V. germanica* and 696 *V. vulgaris*) wasps were captured at nest entrances from 15 nests (6 of *V. germanica* and 9 of *V. vulgaris*). Of the 1059 individuals captured, 564 (224 *V. germanica* and 340 *V. vulgaris*) had crop contents with ≥ 1% w/w. Those carrying liquid with lower concentrations were considered to be water carriers (*V. germanica*: 26 individuals and *V. vulgaris*: 33 individuals), and the rest of the individuals did not have liquid content in their crops (i.e., “empty”, *V. germanica:* 113 and *V. vulgaris:* 323).

#### Crop-content sugar concentration

**Figure 2:**
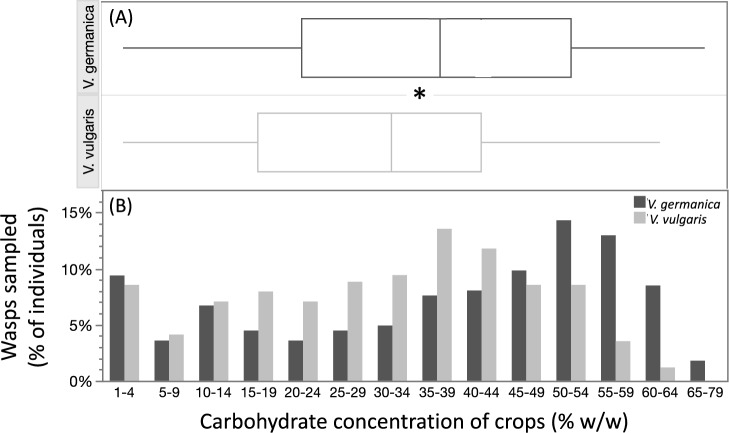
Carbohydrate concentration of crop contents obtained from *Vespula germanica* (n = 224) and *Vespula vulgaris* (n = 340) workers entering nests. (**A**) A statistical difference was found between species, with *V. germanica* having higher concentrations than *V. vulgaris* (*p < 0.05). The left and right boundaries of the boxes indicate the 75th and 25th percentile, respectively. The lines within the boxes mark the mean. Whiskers show 90th and 10th percentiles. (**B**) Sugar concentration of crop contents of *V. germanica* and *V. vulgaris*.

Significant statistical differences in the sugar concentration of crop contents were detected between the species, with *V. germanica* carrying higher concentrations than *V. vulgaris* (*V. germanica* = 37.60 ± 1.32% w/w, n = 224 and *V. vulgaris* = 30.59 ± 0.87% w/w, n = 340 (mean ± s.e); F = 7.31, p = 0.02, d.f = 1) (Fig. 2A). The distribution of sugar concentration of crop contents showed that 57% of *V. germanica* foragers sampled had crop contents of ≥ 50% w/w, while only 23% of *V. vulgaris* foragers had contents above this concentration (Fig. 2B).

**Figure 3:**
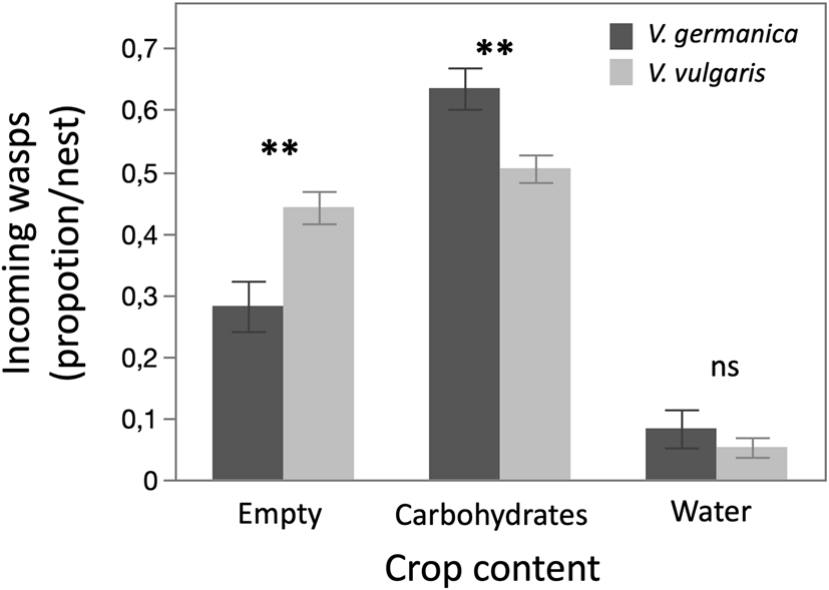
Type of load of foragers of *Vespula germanica* and *Vespula vulgaris* captured with a hand-held battery-operated suction device when entering nests. Captured individuals were classified into “Empty”, “Carbohydrates” and “Water”. Significant differences between species were found in those carrying sugary liquids and those empty. Bars indicate mean values, error bars indicate standard error (*p < 0.05, ns = p > 0.05).

Differences were found in the type of loads carried by both species of yellowjacket wasps. A greater proportion of *V. germanica* wasps were found to carry sugary liquid than *V. vulgaris* (F = 12.4, p < 0.01, d.f. = 1, N = 30). The proportion of wasps returning empty was greater for *V. vulgaris* (F = 12.4, p < 0.01, d.f. = 1, N = 30,), while no differences were found in the proportion of wasps carrying water (F = 3, p > 0.05, d.f. = 1, N = 30) (Fig. 3, Table S3).

#### Nest and nutrition

**Figure 4:**
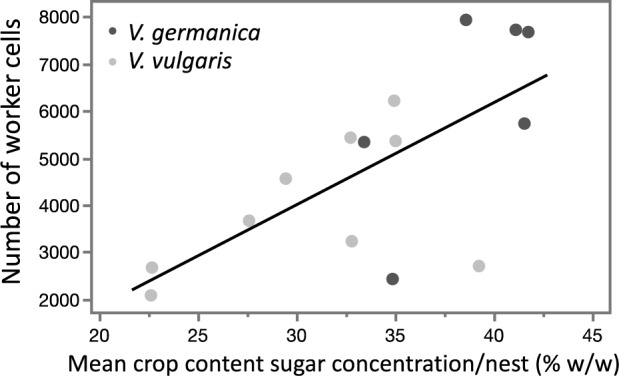
A significant positive correlation was found between the average sugar concentration vs. the final number of worker cells, regardless of the species. (Black line represents the linear fit: y = 228x − 2733; R^2^ = 0.47; *p < 0.05).

A significant positive correlation was found between incoming carbohydrate concentration and the number of total cells (gynes + workers; t = 2.26; p = 0.04, Table S4) and worker cells (t = 2.53; p = 0.03, Table S4) (Fig. 4). No correlation was found between carbohydrate concentration and all other indicators obtained from nests, gynes, and workers (Table S4).

## Discussion

Carbohydrate availability and composition is considered a crucial factor contributing to the ecological success of numerous insect species. For invasive social wasps, carbohydrates are key resources for adult survival, and therefore for nest rapid growth. In this study, we aimed to determine whether variations in carbohydrate sensory responses could modulate foraging patterns of two *Vespula* species present in Patagonia-Argentina, under the hypothesis that sensory differences toward a particular resource could modulate niche partitioning and reduce competition. Our results indicate that responses in laboratory bioassays analysing response towards carbohydrate solutions of increasing concentration, the proportion of responding *V. vulgaris* individuals was consistently and significantly greater than that of *V. germanica* more *V. vulgaris* responded to lower sugar concentrations and when the concentration increased, this species had consistently higher responses*.* This translates into a higher sucrose response threshold in *V. germanica* than that observed for *V. vulgaris,* implying that *V. vulgaris* would be willing to collect sugary liquids of lower concentrations. Field results indicate that the concentration of foraged carbohydrates is higher for *V. germanica*, with 57% of *V. germanica* foragers returning with carbohydrates above 50% w/w, while only 23% of *V. vulgaris* foragers returned to their nests with sugary liquids above this concentration. In spite of these differences in sucrose sensitivity and foraging patterns between species, we only found a positive correlation between sugar concentration and colony size, irrespective of the species. This effect, not large enough to denote differences in final colony sizes between species, was found at the genus level (i.e., *Vespula* spp.) with a clear positive correlation between colony size and average sugar concentration.

Previous studies have shown that responses at a sensory level are fundamental in driving behavior within- and between-species. Research conducted on within-species variations in the perception of relevant resources during the lifespan of an individual is abundant, with results showing it influences the likelihood of initiating certain activities, such as foraging or collecting tasks of specific resources in social insects. It has been observed that bee foragers exhibit lower response levels to sugar resources than pre-foragers, while pollen collectors display significantly lower sucrose response than nectar collectors^38–41^. Similar results have been observed in *V. germanica,* where differences in susceptibility to sucrose were observed between two groups (i.e., foragers had lower thresholds for sucrose sensitivity than pre-foragers); however, the opposite was observed for proteins (i.e., peptone) thresholds^32^. Gustatory response toward carbohydrates imply the integration of gustatory perception and motivation for sugar, ultimately allowing feeding. The response also occurs in other insect species and has therefore been used to investigate different aspects of their sensory behaviors. Examples of this are found in the moths *Agrotis ipsilon*^42^ and *Spodoptera littoralis*^43^, the butterfly *Agraulis vanilla*^44^, social vespids^32^ and ants^31^.

Contrastingly, examples of differences in sensory discrimination that promote distinct behaviors are less abundant than those focused on within-species variations. Some of these studies have shown that sensory differences in closely related species could be an underlying mechanism to niche partitioning thus reducing competition. Our study shows consistent differences in the responses toward carbohydrate solutions between *V. germanica* and *V. vulgaris*, with the former requiring more concentrated sugar solutions for the response to be elicited. These differences in response levels between species were observed to be consistent as of solutions with 3% w/w. This suggests that at a physiological level, the responses in both species are different, with *V. germanica* requiring higher concentrations than *V. vulgaris* to trigger the sucrose-associated response. This difference is maintained throughout the doses applied, with *V. vulgaris* consistently showing higher response levels toward the same sugar concentration than its congener.

In addition, this difference in response was found to correspond with variations in crop loads recorded in our field studies, where the concentration of regurgitated liquid of returning wasps was significantly different between species (*V. germanica* carried higher-concentrated solutions than *V. vulgaris* (37 vs. 30% w/w, respectively)). This suggests a degree of niche separation that could reduce competition and promote coexistence of both species; with *V. germanica* exploiting more concentrated sources, and *V. vulgaris* focusing on less concentrated sources. Pamminger et al.^45^ define nectar concentrations of 65–35% w/w as optimal, 35–20% w/w as adequate, and below 20% w/w as low quality, for pollinators in general. This could explain in part why no larger differences were found between species in the performance parameters measured on individuals and nests at the end of the season. Interestingly no differences were detected in the parameters measured (individual size, nutritional index, and colony size) between species, but a statistically significant effect was found on the final number of worker cells in nests, regardless of the species, driven by sugar concentration. This positive correlation of incoming sugar concentration is significant at the genus level (i.e., *Vespula* spp.) resulting in a possible association between those the concentration of collected carbohydrates, with colony size. More concentrated incoming sugar solutions could mean that wasps are longer-lived and more efficient in terms of foraging trips. Previous studies indicate no differences in crop-content volumes of *V. germanica* and *V. vulgaris*^23^. At equal load volumes, a trip would have more value in terms of calories if the solution were more concentrated, probably impacting in larger final colony size. It's important to note that previous studies have found that, under equal dietary conditions, growth and development rates between colonies of *V. germanica* and *V. vulgaris* for colonies are similar under equal resource-availability regimes^46^. Our results indicate that, despite differences found in incoming sugar solution concentrations, both species appear to collect sugar solutions of “good quality” as defined by Pamminger^45^.

Comparisons between the type of load (sugary liquid, water, or empty) between species show that the proportion of *V. germanica* carbohydrate foragers is higher compared to *V. vulgaris*; while a higher proportion of *V. vulgaris* wasps had no load. This difference was not expected. However, it's important to note that wasps with no loads are probably scouts or wasps carrying solid loads that were lost during the capture process. This suggests that foraging patterns in terms of liquid vs. solid loads could be different between the two species. Further studies on crop contents are needed to establish whether there is compensation for lower liquid carbohydrate qualities with carbohydrates in solid form (e.g. apple lost during captures). Additionally, it is important to mention that other quality criteria (e.g. sugar composition, nectar volume as well as the presence and absence of non-sugar compounds) could also be relevant resource quality parameters that were not contemplated in the present study^36^.

Our study aims at understanding possible underlying mechanisms that could modulate the complexity of colony resource dynamics in a context of close inter-specific competition. In this regard, it's important to mention that additional field studies are required to determine whether the results observed in collection behavior are indeed the result of niche partitioning and not being confounded by other effects driving these differences. For instance, differences in the way that both species handle the sugary liquid once collected, could promote these differences. Detailed studies, aimed at understanding how social vespids handle carbohydrates before and after returning to the colonies are lacking, but previous studies carried out in honeybees report that forager bees can concentrate the nectar load before arriving to the hive via evaporation through the mouthparts^47^ and up to to 80% once stored in the hive^48^. Additionally, more or less concentrated sugar solutions are provided within the hive to foragers depending on whether they are about to set off on a nectar or pollen foraging trip^49^. Even though our study involved only returning foragers, further studies comparing the concentration of sugary liquids at the source and upon arrival to the colony and between the crop contents of incoming and outgoing foragers of both species could help clarifying this.

In this sense, it is still unclear whether the driver for the observed behavior is effectively an innate difference in sugar perception or due to particular experiences acquired due to particular life histories, or *V. vulgaris* carbohydrate foragers could collect water in the same trip while *V. germanica* exclusively collects liquid carbohydrates. Moreover, additional studies to establish effective competition levels need to be carried out by evaluating visiting rates of both species to the array of carbohydrate sources available in the area, in addition to establishing the sugar composition (i.e., sugar types and relative concentrations) of these sources. Despite the questions remaining, our results suggest that competition could be reduced in these closely related invasive social wasp species through sensory differences in their sugar perception levels. Both laboratory and field studies indicate differences in their physiological responses and field foraging behaviours of carbohydrates between *V. germanica* and *V. vulgaris*. The present study suggest that sensory niche partitioning could modulate species coexistence in these social wasps.

### Supplementary Information

Supplementary Tables.

## Data Availability

Data sets generated during the current study are available as electronic supplementary material.
