# Supplementary material for: Sugar responsiveness could determine foraging patterns in yellowjackets
Source: Sci Rep. 2023 Nov 22;13:20448. doi: 10.1038/s41598-023-47819-w (PMC10665408; doi:10.1038/s41598-023-47819-w)
Supplement: Supplementary file 1 — Supplementary Tables. [file 41598_2023_47819_MOESM1_ESM.docx]

# Supplementary material

**Table S1.** Analysis of variance contrasting the proportion of wasps of *Vespula germanica* and *Vespula vulgaris* (species) that positively responded to sugar solutions of different concentrations (trial). Bold letters indicate there were statistical differences. Fit: glmer (response:yes.no.~ Species*trial + (1|id) + (1|nest), data=datos_fluid, family="binomial").

|  | α^2^ | d.f. | **p (>α^2^)** |
| --- | --- | --- | --- |
| Species | 5,63 | 1 | **0,02** |
| trial | 69,05 | 6 | **<0,001** |
| Species:trial | 18,56 | 6 | **<0,01** |

**Table S2.** Post-hoc contrasts between *Vespula germanica* and *Vespula vulgaris* considering the proportion of workers that responded positively to the sucrose solution according to the species. Bold letters indicate there were statistical differences. Fit: emmeans (Model, pairwise~Species|trial).

| **Concentration (% w/w)** | **Proportion responding (mean ± s.e.)** | | **Estimate** | **Std. Error** | **z value** | **P(>\|z\|)** |
| --- | --- | --- | --- | --- | --- | --- |
|  | ***V. germanica*** | ***V vulgaris*** |  |  |  |  |
| 0.01 | 0.46 ± 0.08 | 0.35 ± 0.08 | 0.458 | 0.553 | 0.828 | 0.4076 |
| 0.1 | 0.32 ± 0.08 | 0.29 ± 0.08 | 0.283 | 0.583 | 0.486 | 0.6273 |
| 1 | 0.27 ± 0.07 | 0.47 ± 0.09 | -0,639 | 0.566 | -1.128 | 0.2592 |
| 3 | 0.43 ± 0.08 | 0.74 ± 0.08 | -1.374 | 0.562 | -2.444 | **0.0145** |
| 5 | 0.54 ± 0.08 | 0.74 ± 0.08 | -1.527 | 0.588 | -2.595 | **0.0095** |
| 10 | 0.54 ± 0.08 | 0.82 ± 0.07 | -1.872 | 0.612 | -3.059 | **0.0022** |
| 50 | 0.81 ± 0.07 | 0.97 ± 0.03 | -2.544 | 1.152 | -2.209 | **0.0272** |

**Table S3.** Post-hoc comparisons for the proportion of *Vespula germanica* and *Vespula vulgaris* foragers carrying “water” (% w/w <1), “sugary liquid” (% w/w ≥1) or “empty” measured on *V. germanica* (n=6) and *V. vulgaris* (n=9) nests. Bold numbers highlight statistical differences.

|  | **Proportion of wasps (mean ± s.e.)** | | **Estimate** | **Std.Error** | **z value** | **P(>\|z\|)** |
| --- | --- | --- | --- | --- | --- | --- |
|  | ***V. germanica*** | ***V vulgaris*** |  |  |  |  |
| **Water (% w/w <1)** | 0,08 ± 0,11 | 0,05 ± 0,07 | -0,16 | 0,03 | -4,84 | **<.0001** |
| **Sugary liquid (% w/w ≥1)** | 0,64 ± 0,12 | 0,51 ± 0,09 | 0,15 | 0,03 | 4,34 | **<.0001** |
| **Empty** | 0,28 ± 0,14 | 0,44 ± 0,11 | 0,01 | 0,01 | 0,78 | 0.4344 |

**Table S4.** The effect of incoming carbohydrate concentration (Conc.) on nests and individual parameters were measured on *Vespula germanica* (n=6) and *Vespula vulgaris* (n=9) nests. No significant differences were found between species in most parameters, except for the number of total cells (gyne + worker) and worker cells. Significant effects are highlighted in bold.

|  |  | Full model effect | Conc. | Species | Conc.* Species |
| --- | --- | --- | --- | --- | --- |
| Nest traffic (workers/min) | | F=0.45; P=0.72; d.f.=3 | t=0.24;P=0.81; d.f.=1 | t=-0.06;P= 0.96; d.f.=1 | t=-0.82; P=0.43; d.f.=1 |
| Number of cells | Gyne + Worker | **F=4.01; P=0.04; d.f.=3** | **t=2.26; P=0.04; d.f.=1** | t=0.33; P=0.75; d.f.=1 | t=0.9; P=0.39; d.f.=1 |
|  | Gyne | F=0.95; P=0.45; d.f.=3 | t=-0.01; P=0.99; d.f.=1 | t=0.91; P=0.38; d.f.=1 | t=-1.08; P=0.30; d.f.=1 |
|  | Worker | **F=4.64; P=0.02; d.f.=3** | **t=2.56; P=0.03; d.f.=1** | t=0.14; P=0.87; d.f.=1 | t=1.26; P=0.23; d.f.=1 |
| Nutritional index | Gyne | F=0.68; P=0.58; d.f.=3 | t=-1.39; P=0.20; d.f.=1 | t=-0.77; P=0.46; d.f.=1 | t=-0.53; P=0.64; d.f.=1 |
|  | Worker | F=0.39; P=0.76; d.f.=3 | t=0.44; P=0.67; d.f.=1 | t=--1.00; P=0.34; d.f.=1 | t=-0.07; P=0.94; d.f.=1 |
